# Effects of daily artificial gravity training on orthostatic tolerance following 60-day strict head-down tilt bedrest

**DOI:** 10.1007/s10286-023-00959-5

**Published:** 2023-06-22

**Authors:** J.-N. Hoenemann, S. Moestl, A. E. van Herwaarden, A. Diedrich, E. Mulder, T. Frett, G. Petrat, W. Pustowalow, M. Arz, K. Heusser, S. Lee, J. Jordan, J. Tank, F. Hoffmann

**Affiliations:** 1 German Aerospace Center – DLR, Institute of Aerospace Medicine, Linder Hoehe, 51147 Cologne, Germany; 2 Department of Internal Medicine III, Division of Cardiology, Pneumology, Angiology, and Intensive Care, University of Cologne, Kerpener Str. 62, 50937 Cologne, Germany; 3 NASA JSC KBR Wyle, Houston, TX USA; 4 Laboratory Medicine, Radboud University Medical Center, Geert Grooteplein Zuid 10, 6525 GA Nijmegen, Netherlands; 5 Department of Medicine, Division of Clinical Pharmacology, Autonomic Dysfunction Service, Vanderbilt University, Nashville, TN USA; 6 Head of Aerospace Medicine, University of Cologne, Albertus-Magnus-Platz, 50923 Cologne, Germany

**Keywords:** Orthostatic tolerance, Bedrest, Artificial gravity, Cardiovascular deconditioning

## Abstract

**Purpose:**

Orthostatic intolerance commonly occurs following immobilization or space flight. We hypothesized that daily artificial gravity training through short-arm centrifugation could help to maintain orthostatic tolerance following head-down tilt bedrest, which is an established terrestrial model for weightlessness.

**Methods:**

We studied 24 healthy persons (eight women; age 33.3 ± 9.0 years; BMI 24.3 ± 2.1 kg/m^2^) who participated in the 60-days head-down tilt bedrest (AGBRESA) study. They were assigned to 30 min/day continuous or 6 × 5 min intermittent short-arm centrifugation with 1Gz at the center of mass or a control group. We performed head-up tilt testing with incremental lower-body negative pressure until presyncope before and after bedrest. We recorded an electrocardiogram, beat-to-beat finger blood pressure, and brachial blood pressure and obtained blood samples from an antecubital venous catheter. Orthostatic tolerance was defined as time to presyncope. We related changes in orthostatic tolerance to changes in plasma volume determined by carbon dioxide rebreathing.

**Results:**

Compared with baseline measurements, supine and upright heart rate increased in all three groups following head-down tilt bedrest. Compared with baseline measurements, time to presyncope decreased by 323 ± 235 s with continuous centrifugation, by 296 ± 508 s with intermittent centrifugation, and by 801 ± 354 s in the control group (*p* = 0.0249 between interventions). The change in orthostatic tolerance was not correlated with changes in plasma volume.

**Conclusions:**

Daily artificial gravity training on a short-arm centrifuge attenuated the reduction in orthostatic tolerance after 60 days of head-down tilt bedrest.

**Supplementary Information:**

The online version contains supplementary material available at 10.1007/s10286-023-00959-5.

## Introduction

**Fig. 1:**
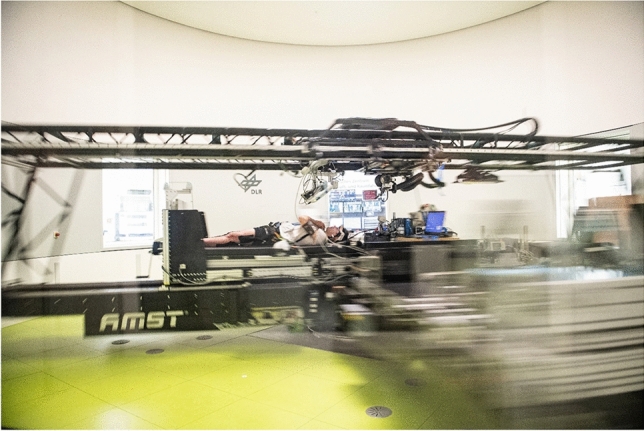
Centrifugation with the short-arm human-centrifuge at the DLR:envihab. Participants were placed with their heads to the center of the centrifuge. The goal was to achieve 1Gz at the center of mass. Instrumentation included three-lead electrocardiogram, oscillometric blood pressure cuff, and video telemetry

Orthostatic intolerance commonly occurs in astronauts returning from space missions [1]. While easily manageable during routine landings on Earth, orthostatic intolerance could have catastrophic consequences during emergency landings or when setting foot on another celestial body. Phenotypically, orthostatic intolerance following spaceflight may present as orthostatic tachycardia, orthostatic hypotension, or neurally mediated syncope [2, 3]. Weightlessness-induced cardiovascular deconditioning and reduced blood volume have been implicated in the pathogenesis of spaceflight-induced orthostatic intolerance [4]. However, altered neurohumoral cardiovascular control and impaired neurovestibular function may also contribute to the response [5]. Because weightlessness is the root cause of all of these changes, artificial gravity through short-arm centrifugation has been proposed as a potential countermeasure [6]. On a short-arm centrifuge, the person is positioned with their head towards the centrifuge axis, resulting in a g-force gradient extending from the head to the feet. (Fig. 1). Unlike other currently employed countermeasures, artificial gravity training could have additional benefits by counteracting cephalad fluid shifts. A persistent cephalad fluid shift predisposes to further pathologies, especially spaceflight-associated neuro-ocular syndrome (SANS), and is not addressed by currently available countermeasures [7]. Head-down bedrest is an established terrestrial model for weightlessness-induced deconditioning and cephalad fluid shifts [8]. Promising effects of artificial gravity on orthostatic tolerance have been described in acute terrestrial studies [9, 10] and in head-down bedrest studies ranging from 5 days up to 60 days [11–13]. Following 5 days of bedrest, intermittent artificial gravity was particularly efficacious but could be less well tolerated. In addition, cardiovascular deconditioning is more pronounced after bedrest [14–16] and after space flight of longer durations [17]. Given that future missions to the Moon and Mars will span longer durations, conducting 60-day simulation studies on Earth may provide a more accurate representation of the challenges faced during actual space flight. Furthermore, whether artificial gravity without additional exercise is a useful multipurpose countermeasure for space travel is unknown. The major aim of this first bedrest study jointly designed and conducted by NASA and ESA (AGBRESA, German Clinical Trials Register DRKS00015677) was to test the efficacy of daily continuous or intermittent artificial gravity training over 60 days of strict head-down tilt bedrest as a potential multipurpose countermeasure for space travel [18]. We tested the hypothesis that artificial gravity training helps in maintaining orthostatic tolerance determined through combined head-up tilt testing with incremental lower body negative pressure. This study, which was particularly well standardized, aimed to provide important insight in efficacy and safety of artificial gravity before considering building such facilities in space.

## Materials and methods

### Participants

Our study is part of the NASA/ESA/DLR 60-day − 6° head-down-tilt bed rest study AGBRESA, which was conducted at the DLR:envihab research facility. Detailed inclusion and exclusion criteria and psychological and medical screening procedures have been described elsewhere [18]. Briefly, men and women who were physically and psychologically healthy, aged between 24 and 55 years, with a body mass index between 19 and 30 kg/m^2^, and non-smokers were potentially eligible. Key exclusion criteria comprised requirements for prescription medications including contraceptives, health conditions that would preclude participation, such as history of cardiovascular disorders including syncope, musculoskeletal, neurological, metabolic or endocrine disturbances, or increased thrombosis risk, among others. Women were required to have a menstrual cycle lasting between 26 and 32 days. We enrolled 24 healthy persons (eight women; age 33.3 ± 9.0 years; BMI 24.3 ± 2.1 kg/m^2^). All subjects provided written informed consent prior to study entry. The study was approved by the North Rhine Medical Association Ethics Committee and prospectively registered on DRKS (German Clinical Trials Register; DRKS00015677).

### Study design and protocol

Study design and standardization measures have been described elsewhere [18]. Briefly, after a 14-day ambulatory baseline period at the :envihab facility, participants underwent 60 days of strict 6° head-down tilt bedrest followed by a 14-day recovery period. Throughout the study, participants were on a highly standardized diet tailored to individual resting metabolic rates with the goal of maintaining body weight within 3% of that measured at baseline. Daily fluid intake from food and beverages was set at 50 ml/kg body weight per day at baseline and during bedrest. Daily sodium intake was adjusted per kg body weight depending on the study phase [18]. Throughout the study, participants were awakened at 06:30 a.m. and lights were turned off at 11:00 p.m.

During the bedrest period, participants were randomly assigned to a control group without countermeasures, daily artificial gravity training through continuous centrifugation over 30 min, or daily artificial gravity training through intermittent centrifugation for six times 5 min with 3-min breaks. Centrifugation induced gravitational forces of 1Gz at the center of mass and 2Gz at the feet. For safety reasons, heart rate was measured continuously by a three-lead electrocardiogram and blood pressure was assessed intermittently with an oscillometric brachial cuff during centrifugation [19]. A control group without exercise has been defined as standard by international space agencies following consultations with independent advisory panels [20].

### Orthostatic tolerance testing

We conducted orthostatic tolerance testing 5 days before the bedrest phase and immediately after 60 days of bedrest in the morning hours after an overnight fast. Every subject was familiarized with the experiment 1 day before we obtained the first measurements. At baseline, subjects walked to the examination room and remained in the supine position for 15 min before the protocol was started. Following the bedrest phase, patients were taken on a stretcher in the head-down position to the tilt table such that orthostatic tolerance testing was the first time participants assumed an upright position. We obtained heart rate through three-lead electrocardiogram and finger blood pressure (Finapres, Ohmeda Medical Instruments, The Netherlands) continuously. We measured oscillometric brachial blood pressure every 2 min. We inserted an antecubital venous catheter for blood sampling. We placed a lower-body negative pressure chamber at the level of the iliac crest. Following instrumentation, participants remained in the supine position for 15 min. We obtained baseline measurements and blood samples in the last 2 min of this period. Then, we tilted the table to 80° head up. After 15 min of head-up tilt, we added incremental lower-body negative pressure of − 10 mmHg every 3 min. We terminated orthostatic tolerance testing when participants wished to abort the test or when presyncope occurred. We defined presyncope as a sudden onset of pallor, blurred vision, lightheadedness, sweating, or nausea, accompanied by a greater than 40% heart rate reduction and/or greater than 40% systolic blood pressure reduction compared to the first 5 min of asymptomatic standing. After the occurrence of presyncope, subjects were tilted back to the supine position and another blood sample was collected. We defined orthostatic tolerance as the time from head-up tilt onset to presyncope. We determined heart rate, systolic blood pressure, and diastolic blood pressure in the supine position, during 15 min of 80° head-up tilt, and with stepwise increase of lower-body negative pressure (LBNP) every 3 min until pre-syncope occurred. At baseline, and immediately after HUT, we averaged 5 min of continuous finger blood pressure and heart rate recordings. We determined finger blood pressure and heart rate during presyncope by averaging up to 60 seconds (if available) before tilting back.

### Vasoactive hormones

After drawing blood, we stored samples on ice until plasma was separated by centrifugation and frozen to − 80° Celsius. Norepinephrine, epinephrine, dopamine, aldosterone, renin, and copeptin were analyzed by an in-house essay developed at Radboud University Nijmegen and validated the method by LC–MS/MS after derivatization with propionic anhydride and subsequent solid-phase extraction (SPE) [21].

### Statistical analysis

Results are reported as mean ± standard deviation. Based on the prospective study design, we used a survival analysis with log-rank (Mantel–Cox) test to assess changes in orthostatic tolerance, the primary objective of this investigation, during head-down tilt bedrest in the centrifugation and control groups. Furthermore, we used an ordinary one-way ANOVA with Tukey's multiple comparisons test to detect possible group differences before and after bed rest. We conducted a mixed-effects analysis with Šidák's multiple comparisons test to analyze hemodynamic and neurohumoral response before and after bedrest. *p* < 0.05 indicated statistical significance. The data supporting the reported results are available from the corresponding author upon reasonable request.

## Results

### Orthostatic tolerance, heart rate, and blood pressure

**Fig. 2:**
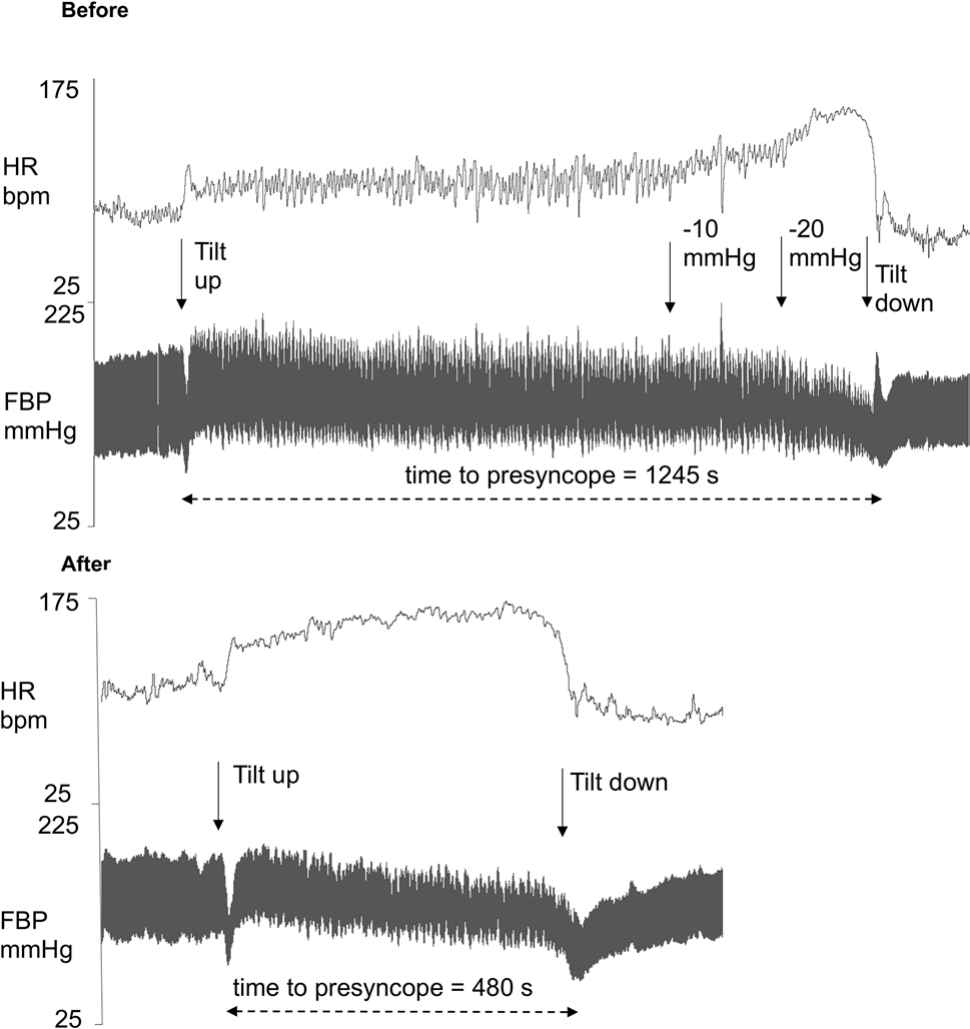
Representative example of orthostatic tolerance testing in one subject before and after bedrest including tracings of continuous ECG and finger blood pressure recordings. Before bedrest, the participant tolerated up to 20 mmHg lower-body negative pressure in addition to head-up tilt before presyncope occurred. After bedrest, heart rate increased more with standing and presyncope occurred before lower-body negative pressure was begun

**Fig. 3:**
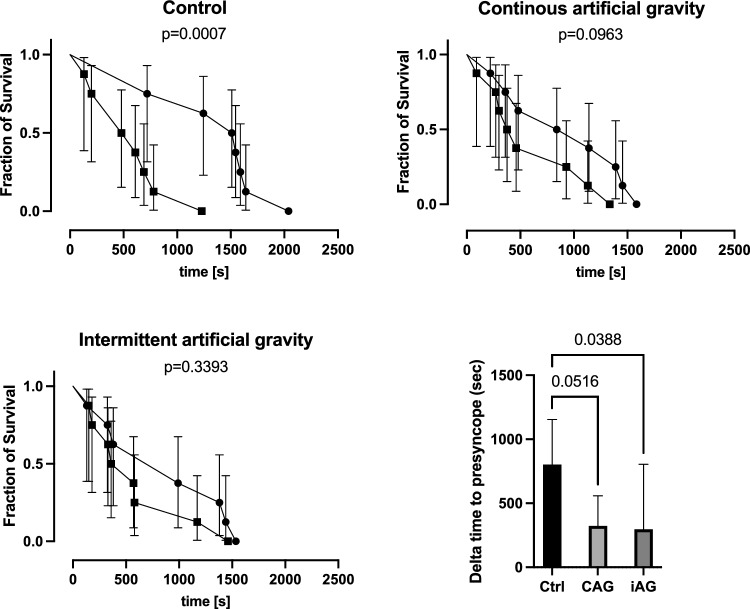
Kaplan–Meier survival curves showing the time to presyncope as surrogate for orthostatic tolerance before and after 60 days of head-down tilt bedrest (mean and 95% confidence interval,* circle*: before bedrest,* square*: after bedrest)

Subjects’ characteristics, plasma volume, and changes in hemodynamics from supine to HUT and at presyncope are listed in Tables 1, 2, and 3 in the supplement. Figure 2 shows representative heart rate and blood pressure tracings during orthostatic tolerance testing before and after head-down tilt bedrest in a participant assigned to the control group. In this participant, heart rate increased more during orthostasis and the time to presyncope was substantially reduced. Figure 3 shows Kaplan–Meier plots illustrating the time to presyncope, the primary readout of this study, in the control group, the continuous centrifugation group, and the intermittent centrifugation group before and after head-down tilt bedrest. At baseline, the time to presyncope was 1376 ± 460 s in the control group, 934 ± 535 s in the continuous centrifugation group, and 896 ± 600 s in the intermittent centrifugation group (main effect, *p* = 0.047). Following 60 days of bedrest, the time to presyncope decreased by 801 ± 354 s in the control group, by 323 ± 235 s in the continuous centrifugation group, and by 296 ± 508 s in the intermittent centrifugation group (*p* = 0.5279 between groups, *p* = 0.0249 interaction bedrest and countermeasure).

**Fig. 4:**
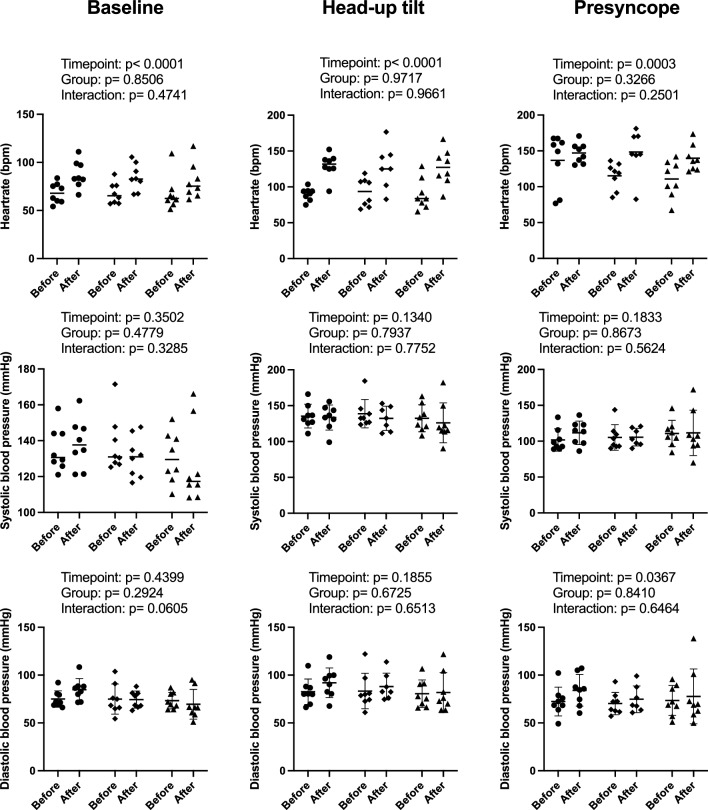
Individual heart rate and finger blood pressure measurements while supine, during early head-up tilt, and at the time of presyncope before and after 60 days of head-down tilt bedrest including adjusted *p* values (mixed-effects analysis with Šidák's multiple comparisons test). The* horizontal bars* indicate the mean value (*circle*: control group,* square*: continuous artificial gravity group,* triangle*: intermittent artificial gravity group). The presented measurements at baseline and during early HUT are mean values of 5 min of continuous finger blood pressure and heart rate recordings. Mean values for presyncope were collected over a period of a maximum of 60 s (if available) before tilting back

Compared with baseline, supine heart rate increased from 68 ± 10 bpm to 87 ± 14 bpm in the control group, from 68 ± 11 bpm to 85 ± 14 bpm in the centrifugation group, and from 68 ± 18 bpm to 80 ± 18 bpm in the intermittent centrifugation group (*p* < 0.0001; Fig. 4). Moreover, heart rate increased more immediately at 80° HUT in all three groups following head-down tilt bedrest (90 ± 9 vs. 130 ± 17 bpm in control group, 92 ± 20 vs. 128 ± 30 bpm with continuous centrifugation, 90 ± 21 vs. 128 ± 25 bpm with intermittent centrifugation; *p* < 0.0001; Fig. 4). At the time of presyncope, heart rate did not differ between groups both before and after head-down-tilt bedrest. Systolic blood pressure was not affected by bed rest and remained unchanged in HUT in all three groups. Diastolic blood pressure was higher at presyncope following bedrest, as it increased from 72 ± 15 to 84 ± 17 mmHg in the control group, from 70 ± 12 to 75 ± 14 mmHg in the CAG, and from 73 ± 16 mmHg to 78 ± 29 mmHg in the iAG (timepoint *p* = 0.0367) (Fig. 4).

### Neurohumoral response

**Fig. 5:**
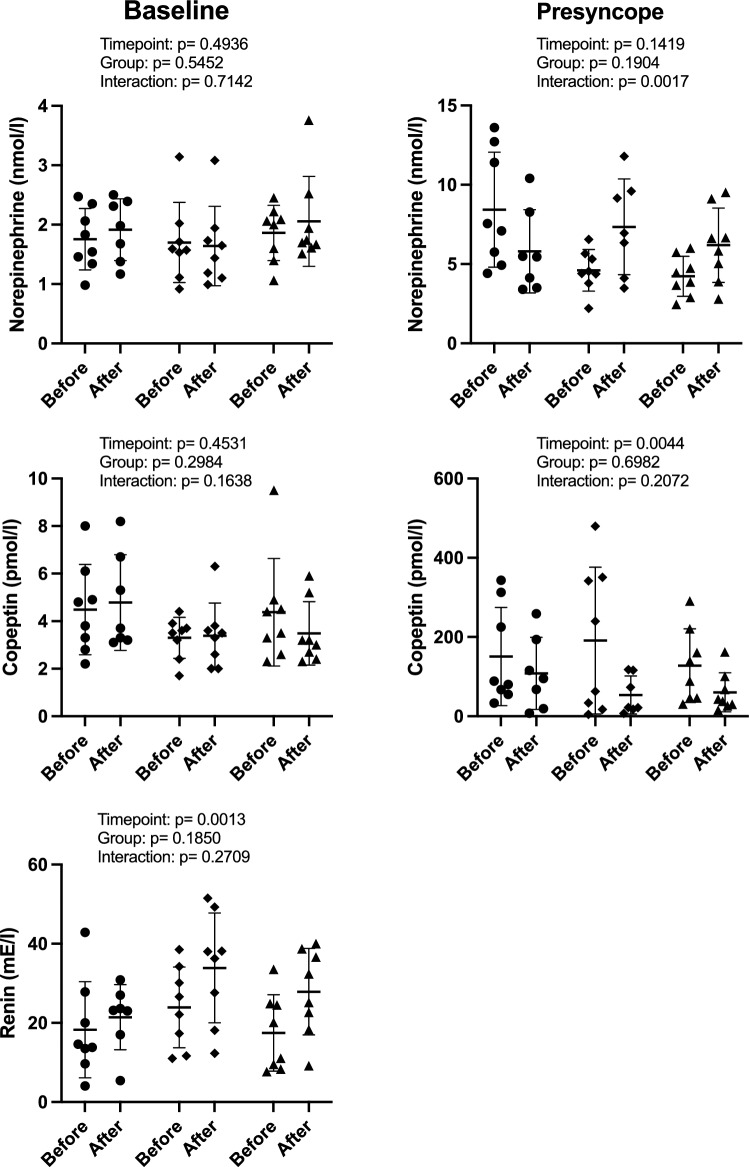
Plasma norepinephrine, copeptin and renin concentrations while supine (*left*) and after presyncope (*right*) before (pre) and after 60 days of head-down tilt bedrest in the continuous centrifugation group, the intermittent centrifugation groups, and the control group. including adjusted *p* values (mixed-effects analysis with Šidák's multiple comparisons test). AG-groups showed higher values of renin following bed rest, which did not lead to an increase in aldosterone plasma concentration (see supplement;* circle*: control group,* square*: continuous artificial gravity group,* triangle*: intermittent artificial gravity group)

Norepinephrine, copeptin, and renin plasma concentrations before and after bedrest in the three study groups are provided in Fig. 5. Supine plasma norepinephrine did not show relevant changes with head-down bedrest in either group. At the time of presyncope, plasma norepinephrine was substantially increased. Norepinephrine at the time of presyncope was reduced in the control group but increased in both intervention groups after head-down tilt bedrest. Supine plasma copeptin concentrations did not show relevant changes with head-down tilt bedrest. Copeptin plasma concentrations were massively increased at the time of presyncope, however, the response was attenuated following bedrest. Supine plasma renin concentrations modestly increased with head-down tilt bedrest without significant differences between study groups (Fig. 5). Epinephrine, dopamine, and aldosterone plasma concentrations before and after bedrest in the three study groups are provided in the online supplement (Tables 4, 5 and 6).

### Plasma volume and orthostatic tolerance

**Fig. 6:**
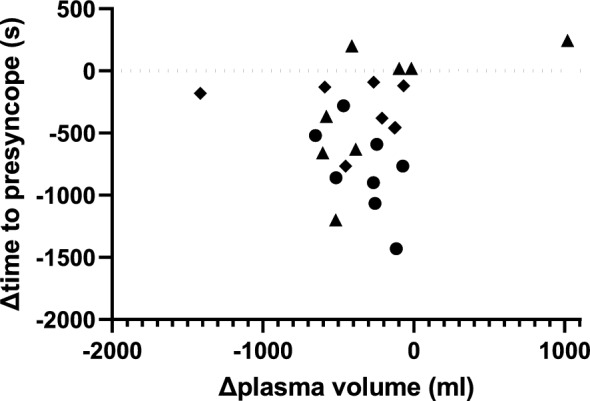
Individual changes in orthostatic tolerance plotter against plasma volume changes (control: *R*^2^ = 0.3223, *p* = 0.1421; CAG: *R*^2^ = 0.03904, *p* = 0.6391; iAG: *R*^2^ = 0.3748, *p* = 0.1067). Linear regression analysis did not reveal significant correlations for each group or for the pooled sample (*circle*: control group,* square*: continuous artificial gravity group,* triangle*: intermittent artificial gravity group)

Plasma volume data determined by carbon monoxide rebreathing have been published previously [22]. We plotted changes in orthostatic tolerance against changes in plasma volume with head-down tilt for all three study groups. The change in orthostatic tolerance was not significantly related to changes in plasma volume overall or for individual study groups (Fig. 6).

## Discussion

The important finding of our study is that daily 30-min artificial gravity training on a short-arm centrifuge with 1Gz at the center of mass ameliorated the reduction in orthostatic tolerance following 60 days of head-down tilt bedrest. However, artificial gravity training did not prevent cardiovascular deconditioning, as supine and upright heart rate was increased in all groups following bedrest. In addition to providing guidance for the development of countermeasures aimed at maintaining orthostatic tolerance during space missions, our findings may have relevance for patients with orthostatic intolerance secondary to deconditioning on Earth.

Head-down bedrest is an established terrestrial model to assess influences of reduced gravity on human physiology. For example, both exposure to real weightlessness in space and head-down tilt bedrest promote orthostatic tachycardia [23, 24]. The methodology has been previously applied to elucidate orthostatic intolerance mechanisms and to test potential countermeasures for space-fight associated orthostatic intolerance [25]. However, influences of head-down tilt bedrest could be confounded by changes in sodium intake, in fluid intake, or in energy balance among other environmental influences [26–28]. In our study, rigorous standardization of the bed rest protocol including controlled sodium, fluid, and energy intake removed these confounders, which is a particular strength [18].

We assessed orthostatic tolerance through head-up tilt testing combined with lower-body negative pressure [29]. The methodology has been successfully applied to show that exercise training, sleeping in the head-up position, increased salt ingestion, and water drinking improve orthostatic tolerance in healthy people and in patients with neurally-mediated syncope [30, 31]. We consider an improvement in orthostatic tolerance by 5 min or more during this test clinically relevant. In the present study, orthostatic tolerance decreased more than 5 min less in both intervention groups compared with the control group. Similarly, daily artificial gravity training elicited through short-arm centrifugation had a beneficial effect on orthostatic tolerance in previous studies, which applied different artificial gravity training protocols [32].

Compared with the early days of astronautical space missions, orthostatic intolerance following return to Earth seems to be less pronounced with current countermeasures. In a recent study in 12 astronauts, none reported orthostatic intolerance following a return to Earth and continuous blood pressure recordings did not reveal major abnormalities. However, orthostatic intolerance and syncope still occur, which could have catastrophic consequences when setting foot on another celestial body. Physical exertion, heat stress, and altered atmosphere conditions during extravehicular activities on the Moon or Mars could conceivably exacerbate orthostatic intolerance.

Orthostatic intolerance following space travel appears to be multifactorial: cardiovascular deconditioning, volume loss, and impaired reflex-mediated sympathetic activation have been implicated [33, 34]. Head-down tilt bedrest reproduces hemodynamic and neurohumoral changes associated with orthostatic intolerance following long-duration space missions [35]. Cardiovascular deconditioning typically occurs following space travel and head-down tilt bedrest [36]. The response was accompanied by left ventricular atrophy in some studies but not in our study participants [37, 38]. Moreover, real weightlessness and head-down tilt bedrest produce reductions in central plasma volume within days [39]. Remarkably, plasma volume changes did not correlate with changed orthostatic tolerance in our study. Finally, impaired norepinephrine release and subsequent vasoconstriction have been observed in astronauts and following head-down tilt bedrest [40, 41]. In the control group, we observed reductions in norepinephrine and copeptin plasma concentrations at the time of presyncope following head-down tilt bedrest. In contrast, the norepinephrine response was augmented in both intervention groups. Copeptin derived from the C-terminal pre-pro hormone containing vasopressin, an established biomarker for vasopressin release, serves as a backup mechanism for blood pressure maintenance when sympathetic counter-regulation fails [42]. It is tempting to speculate that head-down tilt bedrest may have attenuated neurohumoral counter-regulation and that artificial gravity training helped to maintain the response. In any event, artificial gravity training was not sufficient to completely abolish the physiological adaptation promoting orthostatic intolerance. Similarly, a comprehensive review summarizing results from 18 studies testing artificial gravity training during bedrest showed no improvements in plasma volume [43].

### Limitations

Perhaps the most important limitation of our study, as indicated above, is a difference in orthostatic tolerance at baseline. Furthermore, we did not collect blood samples at specific timepoints, but rather at baseline and after presyncope occurrence. We cannot exclude that upright norepinephrine and copeptin measurements are confounded by differences in sampling time-point given the variability in time to presyncope. Moreover, given the complexity and costs of head-down tilt bedrest studies, the number of study participants was relatively small. Combination of head-up tilt and lower body negative pressure is a validated approach to determine orthostatic tolerance, however, active standing, which engages the leg muscle pump, could yield different results [44]. Thus, orthostatic tolerance testing may not always relate to syncope risk be it on Earth, the Moon, or Mars.

## Conclusions

Our study suggests that the artificial gravity protocol applied in our study helped maintain orthostatic tolerance following head-down tilt bedrest. However, the interventions did not suffice to fully prevent cardiovascular deconditioning. Possibly, longer artificial gravity duration or intensity are required. Another approach, which will be tested in future studies, is combining artificial gravity with physical exercise. In addition, our findings highlight the importance of deconditioning in the pathogenesis of orthostatic intolerance in people on Earth. In our study, several previously healthy participants exceeded the diagnostic threshold for the postural tachycardia syndrome immediately following head-down tilt bedrest.

## Supplementary Information

Below is the link to the electronic supplementary material.

Supplementary file1 (PDF 47 KB)
