# Supplementary material for: Effects of daily artificial gravity training on orthostatic tolerance following 60-day strict head-down tilt bedrest
Source: Clin Auton Res. 2023 Jun 22;33(4):401–10. doi: 10.1007/s10286-023-00959-5 (PMC10439060; doi:10.1007/s10286-023-00959-5)
Supplement: Supplementary file 1 — Supplementary file1 (PDF 47 KB) [file 10286_2023_959_MOESM1_ESM.pdf]

Table 1:

Control group

n=8 (6 men, 2 women; Age: 34±8 years; Height: 177±7 cm; Weight: 80±23 kg)

	Supine pre	Supine post	HUT pre	HUT post	Presyncope pre	Presyncope post
Heart rate (bpm)	68±10	87±14	90±9	130±17	137±37	147±14
Systolic blood pressure (mmHg)	135±12	138±14	135±17	133±17	102±16	112±16
Diastolic blood pressure (mmHg)	75±9	85±12	83±13	92±15	72±15	84±17
Plasma volume (ml)	3092±382	2768±267				

Table 2:

Continuous artificial gravity group

n=8 (5 men, 3 women; Age: 32±10; Height: 172±8; Weight: 71±10)

	Supine pre	Supine post	HUT pre	HUT post	Presyncope pre	Presyncope post
Heart rate (bpm)	68±11	85±14	92±20	128±30	115±18	148±33
Systolic blood pressure (mmHg)	138±16	131±11	139±20	132±17	105±18	106±12
Diastolic blood pressure (mmHg)	75±16	74±9	83±19	88±14	70±12	75±14
Plasma volume (ml)	3977±622	2670±444				

Table 3:

Intermittent artificial gravity group

n=8 (5 men, 3 women; Age: 34±11; Height: 174±11; Weight: 71±5)

	Supine pre	Supine post	HUT pre	HUT post	Presyncope pre	Presyncope post
Heart rate (bpm)	68±18	80±18	90±21	128±25	111±25	140±18
Systolic blood pressure (mmHg)	131±14	126±22	132±19	126±28	111±18	112±32
Diastolic blood pressure (mmHg)	73±9	70±16	81±14	82±21	73±16	78±29
Plasma volume (ml)	2894±470	2694±500				

Table 4:

Control group

	Supine pre	Supine post	presyncope pre	Presyncope post
Norepinephrine (nmol/l)	1.75 ± 0.52	1.92 ± 0.52	8.43 ± 3.63	5.81 ± 2.62
Epinephrine (nmol/l)	0.20 ± 0.09	0.32 ± 0.19	1.33 ± 0.84	1.63 ± 1.35
Dopamine (nmol/l)	0.07 ± 0.03	0.08 ± 0.03	0.21 ± 0.13	0.18 ± 0.14
Co-Peptin (pmol/L)	4.49 ± 1.9	4.79 ± 2.01	150.7 ± 123.9	108.3 ± 91.06
Renine (mE/L)	18.29 ± 12.14	21.45 ± 8.24		
Aldosterone (nmol/l)	0.16 ± 0.09	0.12 ± 0.06	0.38 ± 0.26	0.38 ± 0.26

Table 5:

Continuous artificial gravity group

	Supine pre	Supine post	presyncope pre	Presyncope post
Norepinephrine (nmol/l)	1.70 ± 0.68	1.64 ± 0.67	4.61 ± 1.31	7.35 ± 3.02
Epinephrine (nmol/l)	0.18 ± 0.23	0.18 ± 0.1	1.01 ± 0.83	0.82 ± 0.63
Dopamine (nmol/l)	0.06 ± 0.02	0.07 ± 0.02	0.11 ± 0.05	0.16 ± 0.11
Co-Peptin (pmol/L)	3.3 ± 0.87	3.39 ± 1.37	191.2 ± 185.2	53.69 ± 48.11
Renine (mE/L)	23.94 ± 10.19	33.9 ± 13.85		
Aldosterone (nmol/l)	1.08 ± 2.4	0.14 ± 0.08	0.28 ± 0.10	0.33 ± 0.14

Table 6:

Intermittent artificial gravity group

	Supine pre	Supine post	presyncope pre	Presyncope post
Norepinephrine (nmol/l)	1.86 ± 0.46	2.06 ± 0.76	4.23 ± 1.26	6.19 ± 2.34
Epinephrine (nmol/l)	0.17 ± 0.13	0.25 ± 0.11	1.22 ± 0.95	1.33 ± 1.50
Dopamine (nmol/l)	0.07 ± 0.03	0.08 ± 0.02	0.11 ± 0.05	0.16 ± 0.08
Co-Peptin (pmol/L)	4.38 ± 2.27	3.49 ± 1.33	127.9 ± 93.02	60.43 ± 49.19
Renine (mE/L)	17.46 ± 9.65	27.89 ± 10.92		
Aldosterone (nmol/l)	0.18 ± 0.08	0.13 ± 0.08	0.20 ± 0.06	1.13 ± 2.37

Table 1-3: Characteristics and hemodynamics of all subjects listed by groups in supine, early 80° HUT, and at presyncope. The measurements at baseline and early HUT are mean values of five minutes continuous finger blood pressure and heart rate recordings. Mean values for presyncope were collected over a period of maximum 60 seconds (if available) before tilting back.
4-6: Plasma levels of vasoactive hormones obtained in baseline and after presyncope while tilt-table-tesing before and after bedrest (pre and post) in each group. Bed rest did not lead to increased values of norepinephrine in iAG and control, but in CAG. The reduction in plasma volume through bed rest did not increase the response of aldosterone secretion, but renine plasma levels were elevated following HDBR in all groups.
